# Foot contact forces can be used to personalize a wearable robot during human walking

**DOI:** 10.1038/s41598-022-14776-9

**Published:** 2022-06-29

**Authors:** Michael Jacobson, Prakyath Kantharaju, Hyeongkeun Jeong, Jae-Kwan Ryu, Jung-Jae Park, Hyun-Joon Chung, Myunghee Kim

**Affiliations:** 1 University of Illinois at Chicago, Mechanical and Industrial Engineering, Chicago, 60607 USA; 2 LIG Nex1, Seongnam, 13488 South Korea; 3 Korea Institute of Robotics and Technology Convergence, Pohang, South Korea

**Keywords:** Biomedical engineering, Rehabilitation

## Abstract

Individuals with below-knee amputation (BKA) experience increased physical effort when walking, and the use of a robotic ankle-foot prosthesis (AFP) can reduce such effort. The walking effort could be further reduced if the robot is personalized to the wearer using human-in-the-loop (HIL) optimization of wearable robot parameters. The conventional physiological measurement, however, requires a long estimation time, hampering real-time optimization due to the limited experimental time budget. This study hypothesized that a function of foot contact force, the symmetric foot force-time integral (FFTI), could be used as a cost function for HIL optimization to rapidly estimate the physical effort of walking. We found that the new cost function presents a reasonable correlation with measured metabolic cost. When we employed the new cost function in HIL ankle-foot prosthesis stiffness parameter optimization, 8 individuals with simulated amputation reduced their metabolic cost of walking, greater than 15% (p < 0.02), compared to the weight-based and control-off conditions. The symmetry cost using the FFTI percentage was lower for the optimal condition, compared to all other conditions (p < 0.05). This study suggests that foot force-time integral symmetry using foot pressure sensors can be used as a cost function when optimizing a wearable robot parameter.

## Introduction

A below-knee amputation (BKA) is one of the most common types of major amputation worldwide^1,2^, yet it can be difficult to walk easily with an artificial limb^3–5^. As a result, individuals with BKA have expressed the desire for prosthetic devices that reduce the physical effort of walking^6^. One strategy to reduce physical effort is to prescribe a prosthetic foot with stiffness that is personalized to the wearer^7–10^. The stiffness is individually adjusted by clinical experts according to their observations, but this becomes difficult with an increasing number of prosthesis users^11^ along with a shortage of clinical resources in the near future^12,13^. The user’s body weight can also be used to adjust the stiffness^14^; however, recent studies suggest that a weight-based stiffness may not be the most metabolically economic^8^.

Human-in-the-loop (HIL) optimization has been developed for the task of identifying an optimal, personalized parameter that accounts for inter-subject variability in performance^15–18^. HIL optimization has been used to identify a user-specific assistance parameter in a wearable device and thus contributed to reducing physical effort during walking for healthy individuals^15–18^ and simulated amputees^8^. Individuals with BKA present wide inter-subject performance variability^19^. This increase in performance variability may be partially due to differences in residual limb tissue composition, geometry, and intended prosthetic components to be used distal to the socket^20^. Therefore, an individually tuned ankle-foot prosthesis through HIL optimization may improve assistance benefits by accounting for inter-subject performance variability.

**Figure 1:**
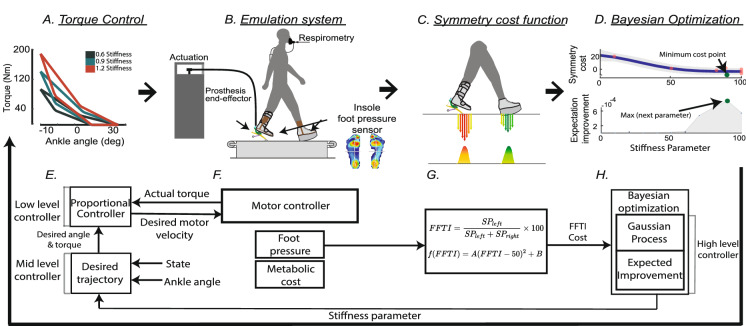
Overview of the human-in-the-loop optimization using the symmetry cost function. (**A**) Mid-level controller: the desired ankle torque was generated depending on ankle angle and stiffness parameter, commanded from the high-level controller. (**B**) Emulation system: the torque was delivered through the ankle-foot prosthesis emulator to a participant while collecting foot pressure and respiratory data. (**C**) Symmetric foot force-time integral (FFTI) cost function: the collected foot pressure was summed in the left ($SP_{left}$) and right side ($SP_{right}$) to estimate the cost of walking. (**D**) High-level controller: Bayesian optimization updated the stiffness parameter to minimize the estimated cost of walking. (**E–H**) Functional block diagram of the system: this block diagram describes how the system works in (**A–D**).

In an HIL optimization scheme that uses metabolic cost as an indicator of physical effort, Bayesian optimization is used due to its sample-efficient and noise-tolerant characteristics^15,21^. Bayesian optimization optimizes a posterior distribution of metabolic cost over the control parameter space to minimize the user’s physical effort. The metabolic cost, used in the cost function, is the energy demand needed to perform a given task^22^. Its measurement, however, is challenging due to slow mitochondrial dynamics and noise in respiratory measurements^23^. As a result, it typically takes at least 5 min to obtain a reasonable estimate per testing condition. This estimation results in increased experimental time; thus, this optimization method has only been performed for walking and partial running^18^ for a healthy individual. In addition, the respiratory measure for this metabolic cost estimation requires an uncomfortable and non-portable physiological sensor. These limitations have led to a search for alternative cost functions to be used with individuals with reduced physical strength, and they should be based on measures that are both time-efficient and comfortable.

Electromyography (EMG) has been used to estimate the metabolic cost of cycling^24^ and joint moments^25^, and this could be leveraged to have a reasonable estimate the metabolic cost of walking^26,27^. Recent advances in measurement systems enabled obtaining additional information such as area of muscle cross-section, length, and velocity of muscle fiber^28^ and calculating accurate metabolic cost. The sensors, however, may be uncomfortable and interfere user’s gait^29^. Another approach is applying machine learning techniques with EMG to estimate the metabolic cost with high correlation. The method demands exponentially higher computational costs^24^. For instance, the estimate of a joint moment with reinforcement learning^25^ requires a relatively long learning time (a maximum of 6 hours), which limits the practical application of EMG on site. Another widely used cost function is a user-based subjective preference. Due to its subjective characteristics, this method often finds different optimum points for each trial, and the optimized assistance tends to show a low correlation with metabolic cost^30,31^.

The ground reaction force can be another candidate to estimate the metabolic cost of walking. Ground reaction force has been used to identify gait characteristics such as deviations from the center of mass^32^, gait symmetry^33^, and the energy relation^34^ in individuals with BKA^35^. In particular, gait symmetry can be a distinguishing feature as healthy individuals present closer to symmetric ground reaction forces between the left and right limbs during walking^36^, compared to BKA^37^. An assistive device can improve the gait symmetry^38^, and such a symmetric gait can reduce the metabolic cost of walking^34^, perhaps by reducing the balance-related effort^39^. These studies suggest that gait symmetry of ground reaction forces (e.g., foot force-time integral (FFTI)^40^) can be used as an objective function for optimizing assistance and can serve as an alternative measure of the metabolic cost. Also, the FFTI information can be quickly obtained using a portable and comfortable foot pressure sensor^33^, which can address another important challenge in HIL optimization, reduced estimation time.

In this study, we hypothesized that the foot force-time integral (FFTI) symmetry could be used to estimate the physical effort of walking as a fast, portable and comfortable measure, and the cost function using the estimated effort can be used in a rapid human-in-the-loop (HIL) optimization scheme. To test this hypothesis, we developed a cost estimation method using the FFTI and evaluated the performance of this algorithm with individuals with simulated amputation^8,41^ using an ankle-foot prosthesis (AFP) emulator as an experimental platform. The cost estimation method was employed in HIL Bayesian optimization of the AFP stiffness parameter to identify subject-specific personalized assistance (Fig. 1). The optimized assistance was compared with the baseline conditions. We expect that the results of this study will broaden the use of HIL optimization with clinically accepted measures such as symmetry and foot pressure. In doing so, the contributions of this study may inform follow-up experiments among individuals with amputation, eventually leading to the design and clinical prescription of prosthetic limbs to reduce walking effort.

**Figure 2:**
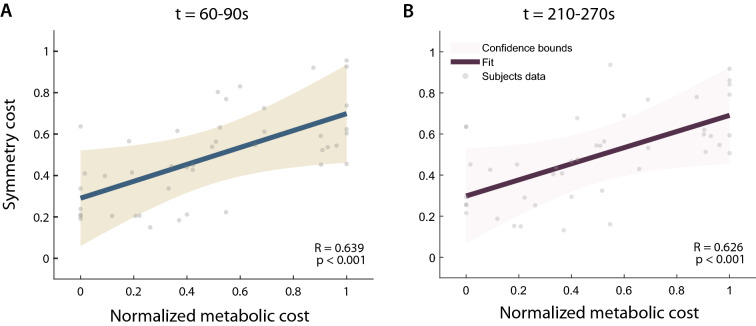
Cost function using symmetric foot force-time integral (FFTI) percentage evaluation results. (**A**,**B**) the correlation between FFTI symmetry cost and normalized measured metabolic cost at time intervals of 60–90 s (**A**) and 210–270 s (**B**). The measured and estimated cost using foot pressure symmetry (gray dots) was fitted with a linear regression (dark curve); also plotted are the confidence bounds of the data (light shade). For both time intervals, each dataset shows a statistically significant and moderately high correlation^42^.

**Table 1:** Optimal stiffness parameter (third row) determined through HIL optimization (second row) for each subject (first row).

Subject	1	2	3	4	5	6	7	8
Optimal normalized stiffness	90.21	80.00	36.16	64.77	20.00	95.00	4.34	9.94
Optimal stiffness parameter	1.67	1.54	0.97	1.34	0.76	1.74	0.56	0.63

The HIL optimization identified the normalized stiffness parameter (second row) and the corresponding stiffness parameter was used to control the device.

## Results

The measured and predicted metabolic cost showed a statistically significant and moderately high correlation^42^(R = 0.64 for time interval 60 - 90 s and R = 0.63 for 210 - 270 s, p < 0.001) (Fig. 2).

**Figure 3:**
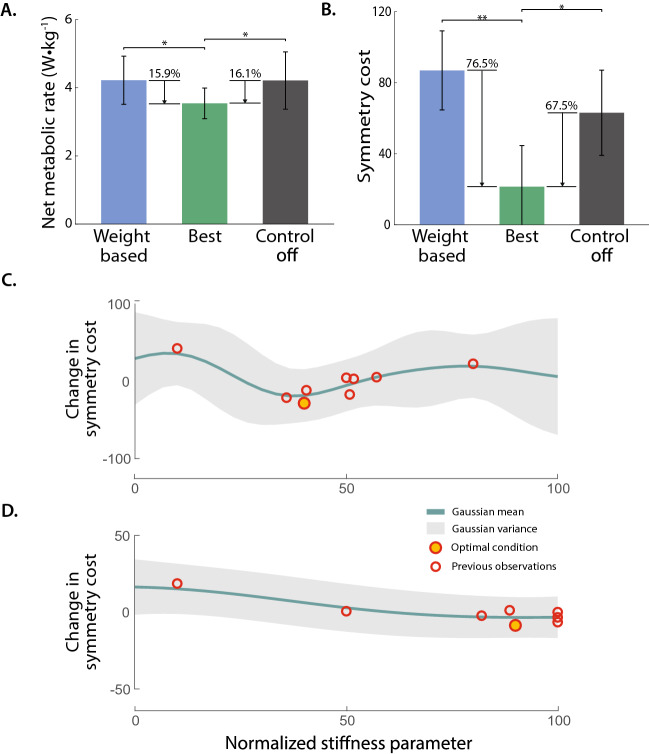
HIL optimization experimental results using the symmetric foot force-time integral (FFTI) percentage. (**A**) Measured metabolic cost of walking, tested in validation trials. Optimized assistance reduced metabolic cost compared to baseline conditions (bars: means; error bars: standard deviations; asterisk: statistical significance (p < 0.05)). (**B**) Symmetry cost during validation trials (double asterisk: statistical significance (p < 0.005)). Optimized assistance presented lower symmetry cost compared to the weight based and control off conditions. (**C–D**) Change in symmetry cost using symmetric FFTI percentage for two representative participants.

The optimal personalized stiffness condition significantly reduced the metabolic cost by 15.9% and 16.1% compared to the weight-based condition and the control-off condition, respectively (paired t-test, p < 0.02) (Fig. 3A).

The symmetry cost was statistically significant and was reduced by 76.5% (paired t-test, p $=$ 0.004) when comparing the optimal personalized stiffness condition to the weight-based condition. The optimal condition also reduced the symmetry cost by 67.5% (paired t-test, p $=$ 0.032) when compared to the control-off condition (Fig. 3B).

The net push-off work energy tended to be maintained for the optimal and weight-based conditions (paired t-test, p = 0.618). The mean values for push-off work were $-0.01 \pm 0.05$ and $0.01 \pm 0.07$ *J*/*kg* for the weight-based and the optimal conditions, respectively.

The perceived effort was statistically significantly reduced for the optimal condition ($12 \pm 2.39$) compared to the weight-based (14 ± 3.07) (paired t-test, p $=$ 0.024), but not compared to the control-off condition ($13 \pm 2.90$) (paired t-test, p $=$ 0.177). Comfort was tended to be increased for the optimal stiffness condition ($6 \pm 2.05$) compared to the weight-based ($5 \pm 1.89$) and control-off ($5 \pm 1.41$) conditions, but not significantly for either condition (paired t-test, p > 0.05).

Bayesian optimization identified the subject-specific optimal stiffness parameters between 0.5 and 1.8 which were scaled between 0 and 100 (Fig. 3C, D, Table 1). The optimization ran for 10 iterations with 60-90s sampling periods to calculate the symmetry cost during each iteration. Each iteration was used to select the next stiffness parameter. The optimization converged within 15 min for all subjects.

## Discussion

In this study, we developed a new cost function using a portable and comfortable mechanical sensor, namely a foot pressure sensor, for use in a human-in-the-loop (HIL) optimization. The cost function using the foot force-time integral (FFTI) also has an important benefit: fast cost estimation. When this cost function was used in the HIL optimization, the optimal stiffness parameter was found within 15 min. The optimized assistance resulted in a lower symmetry cost and reduced the metabolic cost of walking (Fig. 3A). A reduction of approximately 16% in metabolic cost from the optimal stiffness condition to all other conditions demonstrates the efficacy of using a symmetry cost for HIL optimization, as our previous experiment using indirect calorimetry resulted in a metabolic cost reduction of 6% for the optimal stiffness condition for simulated amputees wearing the same device^8^ while maintaining zero push-off work across conditions^43^. The optimal stiffness parameter was varied depending on the participant (Table 1), and each participant presented a subject-specific estimated metabolic cost response surface (Fig. 3C, D). The FFTI-based cost estimation requires a shorter time to find an optimal condition; hence, it has the great potential to enable the application of this personalization method to individuals with limited physical strength. Also, with the portable measurement feature, HIL optimization using a pressure sensor can be used in a natural environment setting.

During the discrete sweep day, we found that the measured metabolic cost using indirect calorimetry and symmetry cost measured with foot pressure showed a statistically significant correlation. The correlation values were 0.64 and 0.63 for 60-90s and 210-270s time intervals using six subjects’ data from Day1, which meant that the correlation was moderately high^42^ and consistent throughout the walking period. When we included Day3 validation data, the correlation values increased to 0.72 for 60-90s and 0.74 for 210-270s, a high positive correlation^42^. Our definition of reasonable correlation values is consistent with the definition from the research guide for medical research^42^ and previous studies for physiological measurement correlation in which values of 0.59-0.64 were used for reasonable correlation^26,44,45^. For instance, changes in integrated EMG and muscle synergy activation were considered to have a high correlation to change in metabolic cost with a correlation coefficient value of 0.64^26^. Our study similarly statistically significantly correlated a physiological signal with another anatomical signal. One of the reasons these values may be acceptable could be due to the noisy and complex nature of physiological data.

The optimized assistance found using the symmetric FFTI and minimizing the symmetry cost helped reduce the metabolic cost of walking. Evidence suggests that an asymmetrical gait caused by varying step length or step frequency could be energetically less optimal^46,47^. In addition, such a gait may result in failure to stabilize the body during the transition between the swing phase and stance phases^48^. Body stabilization to mitigate asymmetrical gait has been shown to increase metabolic cost^34,49,50^. For example, when participants experienced asymmetrical gait while walking on a split-belt treadmill where each belt moved at a different speed, they adapted to the given condition with better symmetric step length along with less metabolic cost^51^. Similarly, our results showed that when participants had a symmetrical gait while walking with a wearable robot (i.e., symmetric FFTI percentage when walking as a result of optimal stiffness), the metabolic cost was statistically significantly lower than with asymmetrical walking.

Our optimization result suggests that HIL optimization using foot pressure can be applied to individuals with amputation as it can find the optimal stiffness parameter in a relatively short time. People with neurological diseases (e.g., stroke, Parkinson’s disease, multiple sclerosis, etc.) and amputation typically present impaired gait, and robotic exoskeletons have been developed to improve their gait performance^37,52,53^. In particular, personalized assistance has been developed to improve gait performance using HIL optimization based on metabolic cost^15,18,54^. Due to noise and slow mitochondrial dynamics^55^, however, the optimization would take a minimum of 24 min, including exploration periods^15,18,21,56,57^. Thus, the optimization method has only been performed for walking and partial running^18^ by an able-bodied counterpart and has been limited regarding the application of patients (e.g., individuals with neurological diseases or amputation having reduced physical strength). This fast estimation (60-90s to calculate the symmetry cost) contributed to reducing the optimization time by 15min, 38% improvement in optimization time, which may enable the use of HIL optimization to the individuals with reduced physical strength.

The data analysis of this study was limited due to the reduced usable Day1 data. The repeated use of the foot pressure sensor caused sensor failure, and we were able to use data from six subjects for the correlation analysis between symmetry cost and metabolic cost (Fig. 2). Prior studies using exoskeleton have conducted experiments and data analysis with 7-10 subjects^15,18,58,59^. Future studies could use more robust sensors, especially considering real-world applications. Additionally, an experiment can be conducted with an increased number of participants to address a potential measurement error with automatic sensor quality check systems.

We hypothesized that the symmetric foot force-time integral would result in metabolically efficient walking. It is possible that a slightly asymmetric gait may be energetically optimal for some subjects. One participant may have an intrinsically asymmetric gait due to long term adaptations to their particular bio-mechanics^60,61^. With our newly developed symmetry cost function, we could shift our ideal location of the global minimum to suit an individual’s intrinsic asymmetrical gait. Future work could explore these intrinsic properties to see how an individual’s neural system may adapt to a perfectly symmetrical assistive condition while walking with an ankle exoskeleton and determine whether providing a slightly asymmetric condition as the optimal parameter could further reduce the metabolic cost of walking.

This study was conducted with only eight male participants. To account for the added height due to the simulator boot and prosthesis, the participants were required to wear a lift shoe. Currently, we have one lift shoe, and to ensure safety, we recruited participants who could comfortably wear the lift shoe. This might limit the applicability of this study’s outcomes. The gait symmetry could be different between males and females^62,63^even if a gender difference in the functional asymmetry is limited^64^. The gait training program helped improve gait symmetry for both males and females^65^, suggesting that the proposed personalization method may be applicable for female participants, but considering the gender differences, it needs to be thoroughly evaluated. Future studies could have multiple lift shoes suitable for diverse demographics and conduct experiments with an increased number of participants.

In this study, we adjusted foot stiffness, a passive parameter, to minimize the symmetry cost in the human-in-the-loop optimization scheme. Evidence suggests that a powered ankle-foot prosthesis could improve metabolic efficiency^66^. Future work can include the active ankle-foot prosthesis control study and compare its effectiveness to the passive prosthesis parameter optimization results.

Further studies are required to test the applicability of symmetric pressure optimization to individuals with amputation. There are numerous differences between individuals with simulated amputation and those with below-knee amputation, such as the training duration for wearing prostheses and sensory-motor control pathways. Perhaps due to these differences, researchers previously observed different outcomes between these populations^53,67^. For the purpose of our study, these differences become less concerning because, similar to a previous study^21^, we used the same factors of mass, height, and alignment, and these factors are unlikely to interact with the summed foot pressure symmetry cost function. While our results are promising, an experiment needs to be conducted with individuals with amputation to draw a conclusion regarding the effect of this HIL optimization using foot force-time integral symmetry. In addition, the investigation of our cost function using foot force-time integral symmetry may also provide insight into the user adaptation to the device and may lead to an efficient method for considering human-robot co-adaptation.

## Methods

We performed an experiment to test the hypothesis that the physical effort can be estimated using foot force-time integral (FFTI) symmetry, and therefore a function of FFTI symmetry can be used to optimize assistance. We conducted walking experiments to evaluate the performance of the physical effort estimation method using FFTI symmetry. The performance of estimating effort with FFTI symmetry was evaluated by conducting a correlation analysis between the measured and estimated metabolic cost, and we assessed the performance of the optimized assistance using the new cost function using FFTI symmetry.

### Prosthesis control

#### Hardware platform

**Figure 4:**
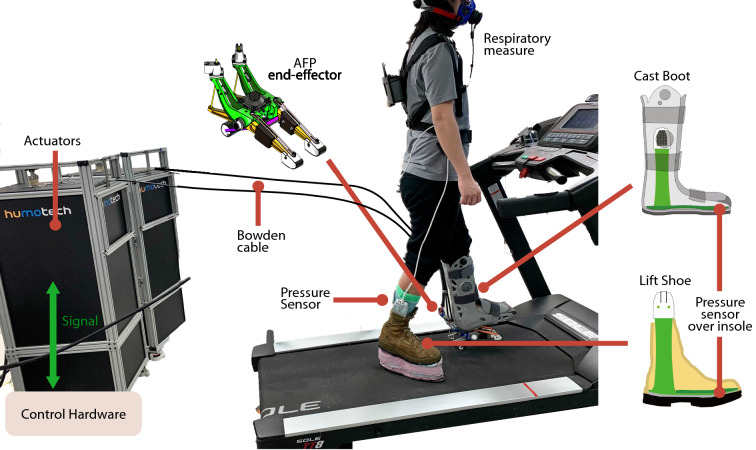
Experimental setup: tethered ankle-foot prosthesis emulator with a cast boot to immobilize the ankle and lift shoe for individuals with simulated amputation. The pressure sensors (green) were located at the insole of the lift shoe and the cast boot. The pressure sensor and respiratory data were used to develop a cost function and to evaluate the performance of the cost function in the HIL optimization scheme.

We used a tethered robotic ankle-foot prosthesis emulator to permit real-time adjustments of free control parameters such as stiffness and net push-off energy^9,39,58,68^. The device provided active plantarflexion torque as a function of ankle angle using the control parameters while users walked with the device (Fig. 4), as described in detail in^56,69^. The rear part of the toe was connected to the two off-board servomotors (Caplex, Humotech, Pittsburgh, PA), which provided power. Control was performed using a real-time control system (Performance Real-Time Target Machine, Speedgoat, Switzerland). The emulator demonstrated performance of 250 Nm plantarflexion peak torque, more than 10 Hz control bandwidth, 17 Hz disturbance rejection bandwidth, and less than 5 Nm error on average in both plantarflexion and dorsiflexion. Those values are well within human ankle torque ranges during typical walking (120 Nm for plantarflexion)^59^. This characteristic enables our device to optimize free control parameters according to continuous biofeedback in the HIL optimization scheme. We also further improved the ankle-foot prosthesis emulator to meet the robustness demand of HIL optimization given an extended experiment duration^56^.

#### Prosthesis control with a free parameter

We developed a controller composed of low-, mid-, and high-level controllers with a free control parameter, namely stiffness (Fig. 1A). The stiffness parameter was selected before an experiment to examine it’s effect on the symmetric FFTI percentage and measured metabolic cost, or it was selected in real-time in the high-level controller during the HIL optimization. The stiffness parameter was used in the mid-level controller for generating a desired trajectory during the stance phase. The low-level controller then conducted linear control to track the desired trajectory.

The low-level controller provided linear torque and position control. The controller calculated the actuation command based on the error between the desired and actual values of ankle angle position and torque. Subsequently, the calculated signals were sent to each Humotech Caplex actuator unit. The desired torque and position, as well as the control mode, were received from the mid-level controller (Fig. 1E, F).

The mid-level controller sent control commands, desired torque and position, and the control mode to the low-level controller based on gait mechanics. For walking, the gait mechanics were divided into a swing and stance phase. The stance phase was further divided into dorsiflexion and plantarflexion. Once there was a transition toward dorsiflexion from the swing phase, torque control was enabled. During the stance phase, the desired ankle torque was commanded based on a piecewise linear function of the ankle angle (Fig. 1A). The piecewise linear function was separated into dorsiflexion and plantarflexion phases where the slope of each phase was scaled by the stiffness parameter^41^. The stiffness parameter was a free control parameter, which was optimized with the high-level controller (Fig. 1D, H). The swing phase acted to enable position control by holding each motor at the desired position. The stiffness parameter, therefore, had no influence during the swing phase.

The high-level controller optimized a control parameter of the mid-level controller, such as the stiffness of the ankle-angle torque curve. In our control scheme, HIL Bayesian optimization performed this high-level control action (Fig. 1D). Bayesian optimization is a sequential design strategy for near-global optimization of a parameterized black-box function and it is a sample-efficient and noise-tolerant method^70^. This method is well suited to optimizing objective functions, which are expensive to evaluate under constraints of noisy physiological signals with a limited time budget^9,71,72^. Bayesian optimization was used here to optimize a posterior distribution of estimated cost using the foot force-time integral over the control parameter space (Fig. 1C, G). In this case, the control parameter, *x*, is the stiffness, which alters the ankle torque curve in the mid-level controller (Fig. 1A).

### HIL Bayesian optimization

The HIL Bayesian optimization was divided into two phases, initialization and optimization. We initialized the HIL Bayesian optimization by evaluating the estimated metabolic cost for three iterations, which correspond to pseudo-randomly chosen stiffness parameters in the range of 0.5 to 1.8 to avoid myopic sampling and premature convergence^15,21,71^. The upper and lower bounds of the stiffness condition were adjusted based on the safety torque limit (180 Nm) and were scaled to 0 to 100 using the min-max method.

After initialization, Bayesian optimization was iteratively performed over two steps using the estimated cost function (Fig. 1C, G): first estimating the posterior distribution of the cost as a function of ankle-foot prosthesis stiffness using a Gaussian process (Fig. 1D, H)^17,73^, and then selecting the next ankle-foot prosthesis stiffness, $x_{n+1}$, to evaluate using the expected improvement (EI) acquisition function (Fig. 1D, H)^17^. This stiffness parameter was sent to the mid-level controller to regulate the torque as a function of ankle angle (Fig. 1A), and assistance was provided to the participant (Fig. 1B). Bayesian optimization was terminated if the experiment time budget, 15 min, was reached or same stiffness parameter was selected by EI for three consecutive iteration by the BO.

The Gaussian process calculated the estimated metabolic cost response surface, which is represented using the mean, $\mu _x$, the covariance, $k(x,x')$. As a standard practice^71^, we used zero mean. For the covarience function, we selected a squared exponential (SE) kernel ($k(x_i,x_j)$) as shown in Eq. (1)^21,56^:

1 $$\begin{aligned} \begin{aligned} k(x_i,x_j) = \sigma ^2_f \cdot e^{(-\frac{(x_i - x_j)^2}{2l^2})} \end{aligned} \end{aligned}$$

where, $\sigma ^2_f$ is the signal variance of the estimated cost (estimated metabolic rate using foot force-time integral symmetry) variance, and *l* is the length scale parameter (stiffness). The $\sigma _f$ and *l* are hyperparameters, and we optimized the hyperparameters at each iteration to maximize the log marginal likelihood of the data, $D = \{{\mathbf {x}},{\mathbf {y}}\};$ where ${\mathbf {x}}$ is the array of stiffness parameters used at iteration ( $1 \ldots n$ ), given by ${\mathbf {x}} = [x_1 \ldots x_n]$ and ${\mathbf {y}}$ is array of the estimated costs evaluated for each stiffness, represented as ${\mathbf {y}} = [y_1 \ldots y_n]$.

The samples of estimated metabolic cost using foot force-time integral symmetry (*f*(*x*)) are assumed to have an additive, independent, and identically distributed noise,

$$\begin{aligned} \begin{aligned} y(x) = f(x) + \epsilon , \epsilon \sim N(0, \sigma ^2_{noise}) \end{aligned} \end{aligned}$$

where $\sigma ^2_{noise}$ is the noise variance and is a hyperparameter. Given the Gaussian process and data, *D*, the posterior estimated metabolic distribution was computed for a stiffness parameter, $x_*$, as $y(x_*) \equiv y^* \sim N(\mu _*, \sigma ^2_*)$,

2 $$\begin{aligned} \begin{aligned} \mu _*&= k^T_*(K + \sigma ^2_{noise} I)^{-1} {\mathbf {y}} \\ \sigma _*&= k(x_*, x_*) + k^T_* (K + \sigma ^2_{noise} I)^{-1} k_* \end{aligned} \end{aligned}$$

where the *K* and *k* were calculated using:

$$\begin{aligned} \begin{aligned} k_*&= [k(x_*,x_1), \ldots , k(x_*, x_n)]^T \\ K&= \begin{pmatrix} k({\mathbf {x}}_1,{\mathbf {x}}_1) &{} \cdots &{} k({\mathbf {x}}_1,{\mathbf {x}}_n)\\ \vdots &{} \ddots &{} \vdots \\ k({\mathbf {x}}_n,{\mathbf {x}}_1) &{} \cdots &{} k({\mathbf {x}}_n,{\mathbf {x}}_n) \end{pmatrix} \end{aligned} \end{aligned}$$

where $x_*$ is discrete stiffness parameter, $x_1 \ldots x_n$ are the stiffness parameter for previous *n* iterations, and $y_1 \ldots y_n$ are estimated metabolic cost using the foot force-time integral from the previous n iterations.

To acquire the next stiffness parameter, we used the expected improvement (EI), which balanced between minimum predictive points and high uncertainty. EI selected the next parameter by calculating the expected reduction in the estimated metabolic cost over the stiffness previously evaluated using Gaussian process posterior distribution using Eq. (3):

3 $$\begin{aligned} \begin{aligned} EI[x_*] = (y_{best} - \mu _*) \cdot CDF(\mu _*) + \sigma _* \cdot PDF(u_*) \end{aligned} \end{aligned}$$

where $y_{best}$ is given as $\min _{1 \ldots n} E[y(x_i)]$, $u_*$ is $(y_{best} - \mu _*) / \sigma _*$, and *CDF* and *PDF* corresponds to cumulative distribution function and probability distribution function of the posterior function (Gaussian process). EI value was set to zero when $\sigma _*$ was zero. The next parameter was then calculated using Eq. (4):

4 $$\begin{aligned} \begin{aligned} x_{n + 1} = argmax_{x_*}(EI[x_*]) \end{aligned} \end{aligned}$$

where the *argmax* function identified stiffness corresponding to the maximum EI value in the parameter range ($x_*$). This newly selected parameter is then passed to the mid-level controller.

### New cost function to estimate metabolic cost

We developed a cost function (*f*(*x*)) to be used in HIL Bayesian optimization based on the symmetry index^74^. Several symmetric indexes have been used with temporal, pressure, and force features^75^. The combination of the ground reaction force and time (force-time integral) had shown the lowest standard deviation compared to other temporal- and force-parameters-only methods^76^. In this study, we calculated the force-time integral using an F-scan insole pressure sensor (Tekscan, MI, USA). We first summed the pressure on each foot to estimate the foot force and then integrated the force through the stance phase. This force-time integral was then used to estimate the metabolic cost of walking.

The F-scan insole sensors were placed at the insole of the lift shoe (right) and the insole of the cast boot (left) (Fig. 4). Each F-scan sensor was connected to the port hub through an ethernet cable. We calibrated both the left and right sensors using the F-scan step calibration function. In this calibration, a subject was asked to stand on the opposite-side leg for 5 seconds and then switch to leg to be calibrated for a remaining 15 seconds. Using the pressure information from the sensor cells and a subject’s body weight, a calibration file was generated. The sensor has 25 sensel per square inch, and we replaced sensors when we saw a 10% drop in the sensing cells. For the real-time streaming, we used F-scan’s Matlab SDK to extract the magnitude of all the sensors in a single frame at 100Hz.

We first obtained the symmetric foot force-time integral (FFTI) percentage, focusing on the limb which is assisted by the ankle-foot prosthesis (AFP), left side:

5 $$\begin{aligned} \begin{aligned} FFTI = \frac{SP_{left}}{SP_{left} + SP_{right}} \times 100 \end{aligned} \end{aligned}$$

where $SP_{left}$ is the summed pressure in the left side and $SP_{right}$ is the summed pressure in the right side. The sum of the pressure (*SP*) for each foot is obtained by adding the pressure in each cell over the stance phase of the gait. Similar to the force-time integral measure^76^, SP captures the sum of force applied during the stance time.

Then, we constructed a cost function based on a symmetry index (SI) with a hypothesis that the metabolic cost would be minimized when a participant loaded equal force between the left and right feet during the stance phase^33,74^ as suggested by the simplified dynamic walking model^46^. Hence, we aim to minimize a function of symmetry index, $SI = {|SP_{left} - SP_{right}|}/({0.5(SP_{left} + SP_{right}))}$:

6 $$\begin{aligned} \begin{aligned} f(x) = \alpha \cdot SI^2 + \beta = A(FFTI - 50)^2 + B \end{aligned} \end{aligned}$$

where $A = \alpha / (25^2)$, and $B = \beta$. The detailed derivation can be found in the supplementary equations.

### Experimental methods

We conducted human subject experiments to evaluate the developed cost function by investigating the correlation between symmetric FFTI percentage and metabolic cost and then by employing the new cost function to optimize the ankle-foot prosthesis (AFP) stiffness parameter in human-in-the-loop (HIL) Bayesian optimization.

#### Participants

Eight healthy male adults (age $28.1 \pm 3.3$ years, weight 74.7 ± 9.1 kg, height $174.8 \pm 6.8$ cm) participated in this study. The experimental protocol was approved by the University of Illinois at Chicago Institutional Review Board. All subjects provided written informed consent in accordance with the Declaration of Helsinki.

For the experiment with individuals with simulated amputation, the ankle-foot prosthesis (AFP) end effector was modified by attaching a cast boot. The intent of walking similarly to amputation was simulated by immobilizing the non-amputated individual’s ankle, which effectively restricts the wearer’s ankle range of motion. The cast boot (Fig. 4) allowed a non-amputated individual with an intact lower extremity to safely interface with the AFP via a pyramidal adapter receptacle attached to the sole of the cast boot^43,67,77^. Subsequently, the non-amputated individual was raised above the opposite limb’s ground reference point, thus requiring the need to wear a lift shoe. The lift shoe is a boot consisting of an elevated sole manufactured from composite foam with a height of approximately 0.1 m.

**Figure 5:**
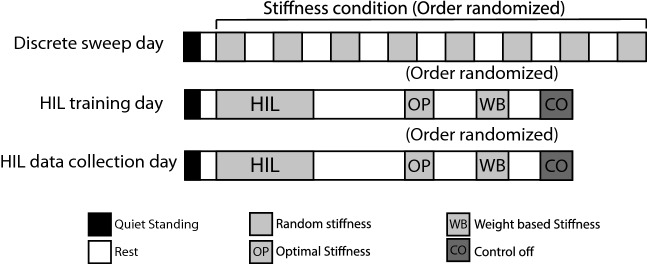
Experimental protocol. The first day was a discrete sweep day when randomly chosen stiffness parameters were tested. The second day was an HIL training day that allows participants to familiarize themselves with the HIL procedure. The final day was the data collection day of the HIL optimization.

#### Experimental protocol

Participants experienced three days of experimental protocol: discrete trials of eight AFP stiffness conditions, HIL optimization training, and HIL optimization data collection (Fig. 5). We provided an additional training day for the three novice participants who wore the device for the first time with the lift shoe^52,78^.

During the discrete trial day (Day1), the neutral angle of the AFP was adjusted to match what was comfortable for the subject and then participants experienced a quiet standing condition for 3 min, which served as a baseline for the metabolic cost and foot pressure. Then, the subjects went through 8 stiffness parameter conditions, from 0.5 - 1.8 in a random order while they walked on a treadmill at a walking speed of 1.25 *m*/*s* for 5 min for each condition with a 5 min sitting break in between. If a participant expressed extreme discomfort and was almost unable to walk, we terminated the condition and excluded the stiffness during the following optimization.

On Day2, the participants experienced a HIL optimization training study to become familiarized with the long experimental time (15 min) while walking on a treadmill during multiple stiffness conditions without a break. After a minimum 24-hour rest, the subjects participated in the same protocol for the data collection on Day3.

For the HIL optimization training (Day2) and data collection days (Day3), participants initially experienced 3 min of standing to measure the base metabolic cost and foot pressure, followed by a 3 min sitting break. The HIL optimization started after 2 min of warm-up, and then the Bayesian optimization occurred over a maximum of 15 min while the subject walked on a treadmill at 1.25 m/s. The Bayesian optimization was said to be converged if three consecutive parameters of the same value were selected. After optimization, the participants experienced a 30 min sitting break. Then, the participants experienced the control-off condition, the weight-based condition, and the optimal condition in a random order for 5 min each. Subjects had a 5 min sitting break in-between each condition and during this break period they were also verbally asked their comfort and perceived effort scores^79^. For the control-off condition, we fixed the motor position; therefore, the participants walked while experiencing compliance from the Bowden cable tether. Characterization of the tether’s compliance has been derived from previous experiments with a similar AFP end-effector^43^. The weight-based condition provided assistance using a stiffness parameter based on the participant’s weight. For this experiment, we selected 1% of subject’s weight^80^. The control-off condition and the weight-based condition served as a baseline^81^ to evaluate the performance of optimized assistance from HIL optimization using the foot force-time integral based cost function.

We collected respiratory rate (Cosmed, Rome, Italy) and foot pressure (Tekscan, Boston, Massachusetts, USA). The obtained respiratory rate was collected with a mask which uses a sampling line to measure the VO_2_ and VCO_2_ output of the subject and encoder to measure flow rate. Foot pressure was measured by an insole placed under the feet of the subject which creates a pressure map from vertical ground reaction forces.^79^. Comfort scores were measured on a scale of 1-10 and perceived effort scores were measured on a scale of 6-20^79^.

### Data analysis

#### Foot force-time integral symmetry and metabolic cost

Using the data from the discrete trial day, we examined our hypothesis that symmetric gait, shown by symmetric foot force-time integral, could be used to minimize metabolic cost. Due to pressure sensor failure, two subjects’ pressure data were not used. We conducted a correlation analysis between estimated metabolic cost using the foot force-time integral and measured metabolic cost^82^. We first calculated the symmetric foot force-time integral (FFTI) percentage using Eq. (5). The symmetric FFTI was further normalized with the min-max method to transform the data range from 0 to 1 (Fig. 2). Outliers were removed with the criterion of three standard deviations from the mean. Then, we obtained the symmetry cost using Eq. (6). The measured, steady-state metabolic cost was calculated by taking the last 2 min data from the respiratory measure and inputting the VO_2_ and VCO_2_ data into the Brockway equation^83^. Then, we normalized the measured metabolic cost for each subject by subtracting the resting metabolic measure obtained from the standing condition and dividing it by the weight of the subject^15,21^. The measured metabolic cost was further normalized in the same manner as the estimated metabolic cost using symmetric FFTI by employing the min-max method and removing outliers.

The correlation between the estimated and measured metabolic costs was examined using a linear Pearson correlation analysis^42^ (Fig. 2). We calculated the p-value and Pearson coefficient, *R*, for two different time intervals: 60 - 90s, and 210 - 270s. The 60 - 90s time period was chosen to represent the minimum possible adaptation period to update the symmetry cost. The 210 - 270s time period was chosen to indicate a consistent measure of the correlation between metabolic cost and symmetry cost throughout the 5 min walking period.

#### HIL optimization using the new cost function

We calculated the normalized steady-state metabolic cost, symmetry cost, and net ankle push-off work for the optimized, weight-based, and control-off conditions from the validation trials. We used the steady-state metabolic cost^83^, divided by body weight and with standing steady-state metabolic cost subtracted. The symmetric FFTI percentage was obtained using the Eq. (5). Then, the FFTI was used to calculate the symmtery cost using the Eq. (6). The net ankle push-off work was calculated by first thresholding the data to extract each step within the stance phase. The threshold used was approximately 10% of the maximum ankle torque^84^. An average of ankle torque and ankle angle was then taken for the total amount of extracted steps. The ankle push-off work was calculated using trapezoidal numerical integration of the ankle torque data with the ankle angle data in time-series as the scalar spacing of each trapezoid^73^.

#### Statistical analysis

We compared the optimal condition to the control-off and the weight-based conditions^18^ for the metabolic cost, symmetry cost, and net push-off work. We first tested normality tests for each condition with the Kolmogorov-Smirnov (KS) test. For the KS test, the alpha level was set to 0.05 and each condition had equal sizes of data. If normality was confirmed, we conducted the paired t-test. The significance levels for statistical analyses were defined at p < 0.05.

## Supplementary Information

Supplementary Information.

## Data Availability

The datasets generated during and/or analyzed during the current study are available from the corresponding author on reasonable request.
