# Supplementary material for: Foot contact forces can be used to personalize a wearable robot during human walking
Source: Sci Rep. 2022 Jun 29;12:10947. doi: 10.1038/s41598-022-14776-9 (PMC9243054; doi:10.1038/s41598-022-14776-9)
Supplement: Supplementary file 1 — Supplementary Information. [file 41598_2022_14776_MOESM1_ESM.pdf]

Supplementary Equations

Derivation of the cost function using symmetric index

The symmetric index for walking is given as follows:

$$SI = \frac{|X_l - X_r|}{\frac{1}{2}(X_l + X_r)}$$

where X is a gait-related parameter such as stance time, step length, or foot force-time integral (FFTI). We obtained the FFTI using the sum of pressure (SI) during the stance phase:

$$SI = \frac{|SP_{left} - SP_{right}|}{\frac{1}{2}(SP_{left} + SP_{right})}$$

where SP_{left} and SP_{right} are the sum of pressure of the left and right foot, respectively. Then, we obtained a cost function by squaring each side and multiplying and adding constants:

$$f(x) = \alpha \cdot 4 \cdot \left(\frac{|SP_{left} - SP_{right}|}{SP_{left} + SP_{right}} \right)^2 + \beta$$

Simplifying further,

$$\begin{aligned}
f(x) &= \alpha \cdot 4 \cdot \left(\frac{|SP_{left} - SP_{right}|}{SP_{left} + SP_{right}} \right)^2 + \beta \\
&= \alpha \cdot 4 \cdot \left(\frac{SP_{left}}{SP_{left} + SP_{right}} - \left(1 - \frac{SP_{left}}{SP_{left} + SP_{right}} \right) \right)^2 + \beta \\
&= \alpha \cdot 16 \cdot \left(\frac{SP_{left}}{SP_{left} + SP_{right}} - \left(\frac{1}{2} \right) \right)^2 + \beta \\
&= A \cdot \left(\frac{SP_{left} \times 100}{SP_{left} + SP_{right}} - 50 \right)^2 + B
\end{aligned}$$

where, A, B are the coefficients of the function defined by $A = \alpha/(25^2)$, $B = \beta$. We obtained the coefficients A and B to minimize the mean square error between the measured metabolic cost from the respiratory measure and estimated metabolic cost using symmetric FFTI percentage data.
